# Biomolecular Dynamics of Nitric Oxide Metabolites and HIF1α in HPV Infection

**DOI:** 10.3390/biom14091172

**Published:** 2024-09-18

**Authors:** Clara Matei, Ilinca Nicolae, Madalina Irina Mitran, Cristina Iulia Mitran, Corina Daniela Ene, Gheorghe Nicolae, Simona Roxana Georgescu, Mircea Tampa

**Affiliations:** 1 Department of Dermatology, ‘Carol Davila’ University of Medicine and Pharmacy, 020021 Bucharest, Romania; matei_clara@yahoo.com (C.M.); srg.dermatology@gmail.com (S.R.G.); dermatology.mt@gmail.com (M.T.); 2 Department of Dermatology, ‘Victor Babes’ Clinical Hospital for Infectious Diseases, 030303 Bucharest, Romania; drnicolaei@yahoo.ro; 3 Department of Microbiology, ‘Carol Davila’ University of Medicine and Pharmacy, 020021 Bucharest, Romania; 4 Departments of Nephrology, ‘Carol Davila’ University of Medicine and Pharmacy, 020021 Bucharest, Romania; corina.ene@umfcd.ro; 5 Department of Nephrology, ‘Carol Davila’ Nephrology Hospital, 010731 Bucharest, Romania; 6 Faculty of Psychology, Babeș-Bolyai University, 400347 Cluj-Napoca, Romania; nicolaengheorghe@gmail.com

**Keywords:** HPV, warts, nitric oxide metabolites, hypoxia, HIF1α

## Abstract

Introduction: Viral infections cause oxygen deprivation, leading to hypoxia or anoxia in certain tissues. The limitation of mitochondrial respiration is one of the major events during hypoxia that induces alternative metabolic activities and increased levels of certain biomolecules such as nitric oxide (NO) metabolites. In this study, we aimed to investigate the role of NO metabolites and hypoxia in HPV infection. Materials and Methods: We included 36 patients with palmoplantar warts and 36 healthy subjects and performed serum determinations of NO metabolites (direct nitrite, total nitrite, nitrate, and 3-nitrotyrosine) and HIF1α, a marker of hypoxia. Results: We found elevated serum levels in NO metabolites and HIF1α, and decreased direct nitrite/nitrate ratios in patients with warts versus controls. Additionally, we identified statistically significant positive correlations between NO metabolites and HIF1α levels, except for 3-nitrotyrosine. Conclusions: Our findings show that HPV infection causes hypoxia and alterations in NO metabolism and suggest a link between wart development and cellular stress. Our research could provide new insights for a comprehensive understanding of the pathogenesis of cutaneous HPV infections.

## 1. Introduction

Nitrosative stress was defined as the imbalance between nitrosants and antioxidants, in favor of the former. Although the concept of nitrosative stress was described several decades ago, many unknowns persist regarding the mechanisms leading to its occurrence. In recent years, attention to nitrosative stress has increased significantly, and substantial progress has been made in this field. Elevated levels of pro-oxidants have been identified in numerous conditions, but it is still unclear whether these high levels are involved in disease onset or are a result of the pathogenic mechanisms specific to the respective condition [1,2].

**Figure 1:**
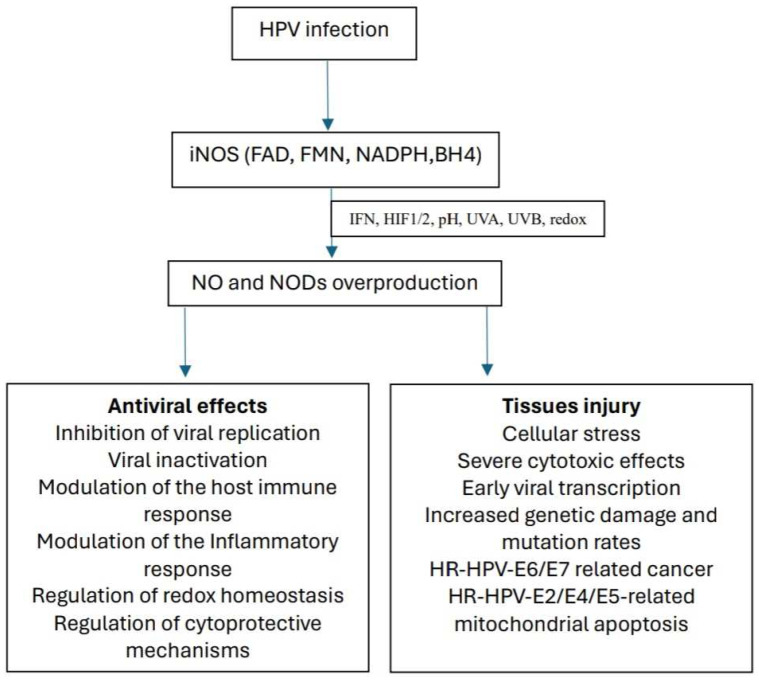
Positive and negative effects of NO/NODs in HPV infection. HPV—human papillomavirus; HR-HPV—high risk human papillomavirus; LR-HPV—low risk human papillomavirus; iNOS—inducible nitric oxide synthase; IFN—interferon; HIF1/2—hypoxia-inducible factor 1/2; NO—nitric oxide; NODs—nitric oxide derivates; FAD—flavin adenine dinucleotides; FMN—flavin mononucleotides; BH4—tetrahydrobiopterin; NADPH—reduced nicotinamide adenine dinucleotide phosphate.

Nitric oxide (NO) is a molecule produced ubiquitously in mammalian cells. NO synthesis is induced by cytokines and endotoxins in endothelial cells, neuronal cells, fibroblasts, platelets, polymorphonuclear leukocytes, and keratinocytes [3,4,5]. In the skin, three NO-synthases (endothelial—eNOS, neuronal—nNOS, and inducible—iNOS) were described that are responsible for the conversion of arginine and O_2_ (in the presence of NADPH, FAD, FMH, and BH4−) into NO and citrulline [5,6,7]. In epithelial cells, constitutively expressed NOS (eNOS, nNOS) generate small amounts of NO, over a relatively short period of time [8,9,10]. In contrast, iNOS produces large amounts of NO over a prolonged period when oxygen-dependent NO is compromised. Excessive production of NO in the skin, insufficiently controlled by iNOS, leads to tissue damage and accelerated synthesis of bioactive nitric NO derivatives (NODs), such as S-nitrosylated proteins (RSNO), N-nitrosamines, metal nitrosyls (RNNO), nitrites (NO_2_^−^), and nitrates (NO_3_^−^). NODs contribute to the overall bioavailability of NO, under conditions of hypoxia, acidosis, exposure to UVA/UVB, and altered oxidant/antioxidant status [3,8,10,11,12] (Figure 1). In addition to the enzymatic NO synthesis from L-arginine, it has been found that a significant amount of NO in the skin may also be released by non-enzymatic pathways from higher nitrogen oxides such as nitrite or nitrate in the reaction of photolysis [13]. Recent studies indicate high levels of nitrosative stress in many skin disorders. It appears to be involved in chronic inflammatory skin diseases such as psoriasis, atopic dermatitis, etc. [14,15]. At the site of inflammation, inflammatory cells can release various reactive nitrogen/oxygen (ROS/RNS) species that exert harmful effects on tissues, leading to the development of nitrosative/oxidative stress. In turn, RNS/ROS can act as secondary messengers and activate various signaling pathways. NO is essential for numerous skin functions, including the proliferation of keratinocytes and fibroblasts, collagen production, and immune response. However, alterations in NO signaling pathways have important consequences on skin homeostasis [16].

Many studies have reported that NO plays a direct or indirect role in the pathology of viral infections such as severe acute respiratory syndrome coronavirus (SARS-CoV-2), human influenzas A and B (including H1N1), human rhinovirus, Crimean Congo hemorrhagic fever virus, vesicular stomatitis virus, human papillomavirus (HPV), herpes simplex virus-1 (HSV), vaccinia virus, porcine circovirus type 2 (PCV2), respiratory syncytial virus (RSV), hepatitis C and B virus infection (HCV, HBV), coxsackievirus3 (CVB3), borna disease virus, encephalitis virus, molluscum contagiosum virus, human immune deficiency virus (HIV), rabies virus, and Sendai virus [17,18,19,20,21,22].

NO is defined as a molecular cofactor in HPV infection (Figure 1). In HPV-infected cells, the balance between the beneficial and harmful effects of NO depends on the type of HPV, NO concentration, cellular microenvironment, rate of formation, and degradation of NO [17,23,24,25]. There are more than 200 HPV subtypes that are categorized into two primary groups: low-risk (LR—HPVs) and high-risk (HR—HPVs) types, based on the potential of the virus to cause malignant transformation. HPV is associated with various skin and mucosal lesions, benign and malignant [26,27]. HR-HPVs promote an inflammatory response, and it is known that persistent inflammation and oxidative stress are associated with tumor progression. The main mechanisms by which HPV induces the development of oxidative stress-mediated neoplasia include dysregulation of mitochondrial function, DNA alteration by ROS, and suppression of enzymatic antioxidant activity. One of the main consequences is that the apoptosis induced by ROS is suppressed in HPV-infected cells [28]. HPV infection can persist and lead to cancer by incorporating its DNA into the host’s genome. Two early viral proteins, E6 and E7, possess the ability to promote cancer development. In vitro studies showed that HR-HPVs are particularly able to persist in human keratinocytes [29,30]. The most common cutaneous lesions related to HPV infection are palmoplantar warts, which are benign lesions. Warts are more common in young men and immunocompromised people, and in the case of the latter, there is a risk of developing squamous cell carcinoma by reactivating the virus in a latent state [31]. The transmission of the infection occurs through direct contact in the context of the existence of microlesions at the skin level or more rarely indirectly through contaminated objects, for example through the utensils used in nail salons [21,23,26]. There are safe and highly effective vaccines available to prevent HPV-related disorders. The bivalent and quadrivalent HPV vaccines have shown considerable effectiveness in preventing HPV infections after being included in vaccination programs [32,33].

Many reports have shown that a hypoxic environment in the skin affects dermal and epidermal morphogenesis, metabolic reprogramming of fibroblasts and keratinocytes, cell migration and proliferation, redox homeostasis, composition and organization of cutaneous lipids, induction of hypoxia-associated genes (angiogenic, metabolic, antioxidant genes) [4,5].

Chronic hypoxia has been shown to modulate NO responses in various cellular models, but the relationship between hypoxia and NOS regulation in humans has been partially studied [34]. The role of NO in the pathogenesis of viral infections is partially understood. We found that there was no previous approach that focused on investigating the NO—hypoxia axis in HPV infection. In this study, our aim was to investigate the role of NO metabolites (nitrites, nitrates, and 3-nitrotyrosine—a specific biomarker for oxidative modifications mediated by reactive nitrogen species) and hypoxia-inducible factor 1 subunit alpha (HIF1α), a marker of hypoxia, in HPV infection. Understanding the role of these biomolecules in HPV pathogenesis could represent an important step in the development of novel biomedical strategies.

## 2. Materials and Methods

### 2.1. Study Participants

**Figure 2:**
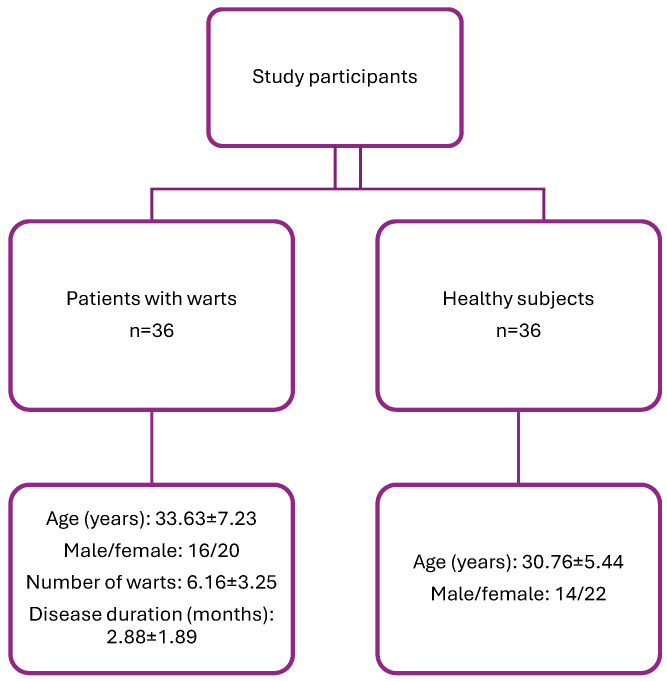
Study participant characteristics.

We conducted an observational case–control study on 36 patients with palmoplantar warts and 36 age (median = 34 years and 31 yeras, respectively, *p* = 0.061) and sex (*p* = 0.63) -matched healthy subjects (Figure 2). The diagnosis of warts was established on clinical aspects. A characteristic sign is the punctiform bleeding that occurs when scraping the lesion, by damaging the capillaries in the dermal papillae, more frequently observed in the case of plantar warts. The main differential diagnoses are actinic keratoses, calluses, seborrheic keratoses, and palmoplantar keratoderma, and sometimes differential diagnosis with squamous cell carcinoma may be necessary [35]. When the clinical aspect was not clear, a histopathological examination was performed to confirm the diagnosis. All subjects included in the study signed a written consent. The study was approved by the Ethics Committee of “Victor Babes Infectious and Tropical Diseases Hospital” (13050/31 July 2017). We quantified the number of lesions and disease duration (the period between disease onset and blood sampling) for each patient.

For the inclusion of participants in the study, the following inclusion and exclusion criteria were used:

Inclusion criteria: age over 18 years, no systemic conditions, appropriate nutritional status, and no treatment of warts.

Exclusion criteria: smokers, alcohol or drug users, pregnant women, treatment with corticosteroids, anti-inflammatory drugs, immunosuppressive drugs, and vitamins. Smokers and alcohol consumers were excluded from the study regardless of whether they have quit this habit. Alcohol and smoking are known to induce long-term changes that will affect the redox status of these patients. Patients who had been treated with anti-inflammatory drugs, corticosteroids, etc., in the last 3 months were excluded from the study.

### 2.2. Sample Collection

Venous blood samples were drawn in a fasting state (7 mL of blood collected). All samples were then centrifugated at 6000× *g* for 10 min. Any unsuitable samples (those that were hemolyzed or lactescent) were excluded. The remaining samples were processed immediately or stored at −80 °C.

### 2.3. Laboratory Determinations

Nitrites and Nitrates. The measurement of the serum levels of NO metabolites (nitrates and nitrites) was performed through the Griess method, which is a colorimetric assay (CAYMAN CHEMICAL CAY 780001, Ann Arbor, MI, USA) [36]. This is a sensitive and specific reaction, which is widely used for nitrate/nitrite assessment. First, the nitrate is converted to nitrite, in a reaction catalyzed by nitrate reductase. Subsequently, the nitrite turns into a deep purple azo compound (a chromophore) under the action of Griess reagents. In the end, we determined the NO_2_^−^ concentration through a photometric measurement. We read the result at a wavelength of 540 nm and employed a TECAN analyzer (GmbH, Grodig, Austria). This method allowed us to measure the levels of direct nitrite, total nitrite, nitrate (the difference between the levels of total nitrites and direct nitrites), and direct nitrite/nitrate.

3-Nitrotyrosine. Circulating 3-nitrotyrosine was measured using an immunoenzymatic method (Cell Biolabs Inc STA305 OxiSelect™ Nitrotyrosine ELISA, San Diego, CA, USA) [37], with sensitive detection down to 10 nM of nitrated BSA provided as a positive control. The results were expressed in μmol/L. A semi-automatic TECAN analyzer (GmbH, Grodig, Austria) operating at a wavelength of 450 nm was used for the colorimetric evaluation of the final product.

HIF1α. The measurement of the serum levels of HIF1α was performed using the ELISA technique (MyBioSource, San Diego, CA, USA) [38]. The microwells were precoated with primary antibodies targeting HIF protein and incubated with serum samples. HIF protein was bound by the coated antibody. Then a second antibody targeting the bound HIF protein was introduced into the reaction and an HRP substrate, to produce color change. For the colorimetric evaluation of the final product the semi-automatic TECAN analyzer, GmbH, Grodig, Austria was employed (wavelength of 450 nm).

ELISA was made in polystyrene plates with 96 wells. The ELISA technique is based on the principle of enzymatic labeling of the antigen/antibody. At a later stage, the enzyme substrate is added, and a color reaction takes place with a change in the optical density of the medium, which can be quantified. Color intensity provides information about the level of the marker that is determined in the sample. The enzyme used was horseradish peroxidase (HRP). It is important to use a stable enzyme with a good ability to bind to the substrate.

### 2.4. Statistical Analysis

We performed the comparison of the two groups included in our study, patients with warts and healthy subjects, using the Wilcoxon test. The normality of data was evaluated with the Kolmogorov–Smirnov test. The correlation between pairs of two variables was assessed by Spearman’s correlation coefficient (rho). *p*-value < 0.05 was established as statistically significant.

## 3. Results

The characteristics of the subjects included in the study are presented in Figure 2.

**Figure 3:**
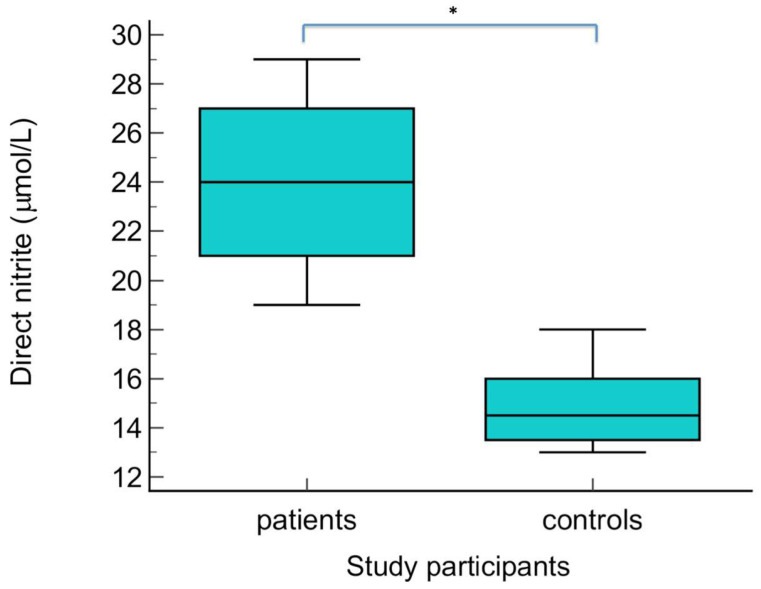
Serum levels of direct nitrite in patients with warts and controls. The graph illustrates the distribution of the dataset, showing the median, interquartile range (middle 50% of the data), minimal, as well as maximal values; * indicates statistically significant difference compared to the control group, *p* < 0.01.

**Figure 4:**
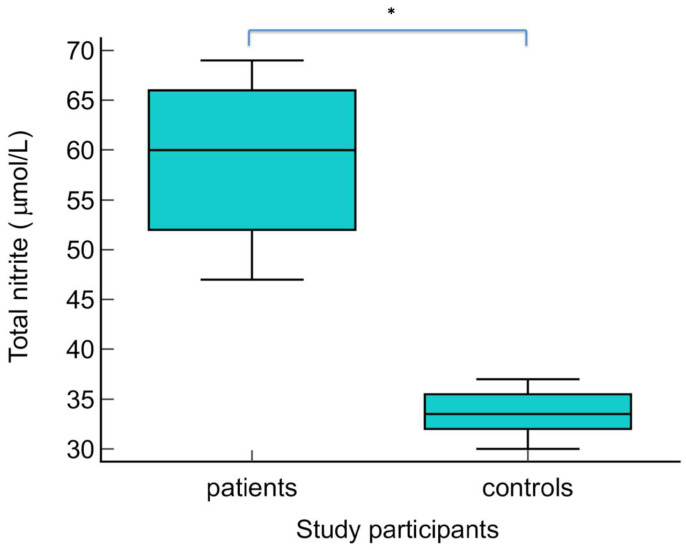
Serum levels of total nitrite in patients with warts and controls. The graph illustrates the distribution of the dataset, showing the median, interquartile range (middle 50% of the data), minimal, as well as maximal values; * indicates statistically significant difference compared to the control group, *p* < 0.01.

**Figure 5:**
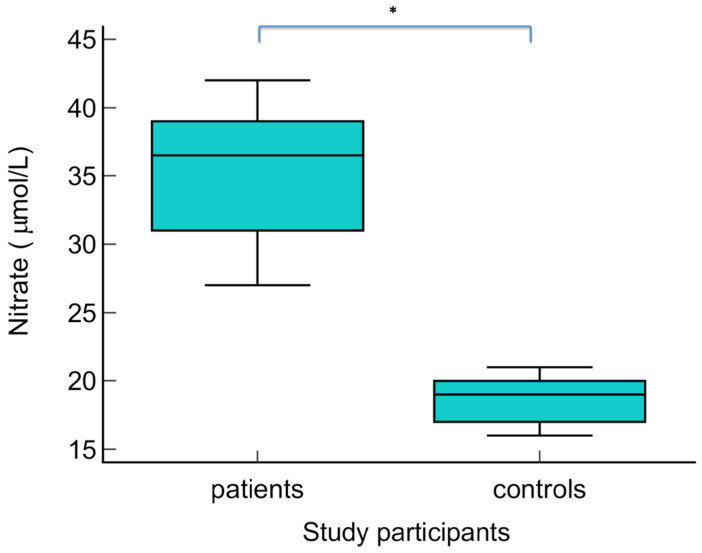
Serum levels of nitrate in patients with warts and controls. The graph illustrates the distribution of the dataset, showing the median, interquartile range (middle 50% of the data), minimal, as well as maximal values; * indicates statistically significant difference compared to the control group, *p* < 0.01.

**Figure 6:**
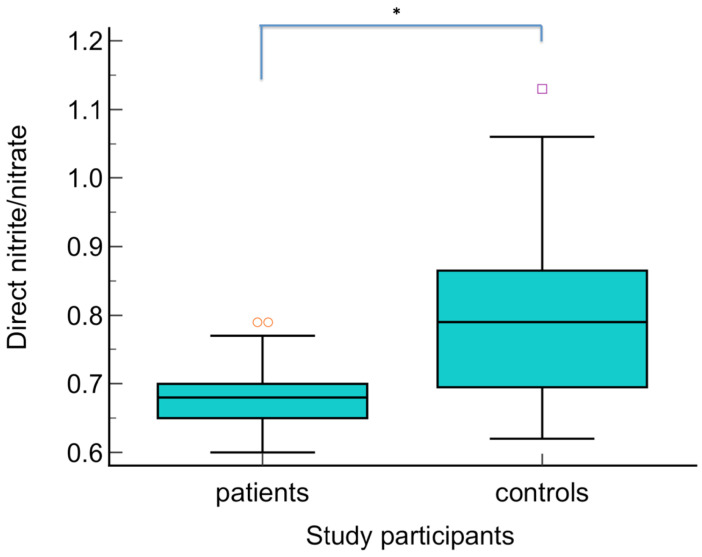
Direct nitrite/nitrate ratio in patients with warts and controls. The graph illustrates the distribution of the dataset, showing the median, interquartile range (middle 50% of the data), minimal, as well as maximal values and outliers; * indicates statistically significant difference compared to the control group, *p* < 0.01.

**Figure 7:**
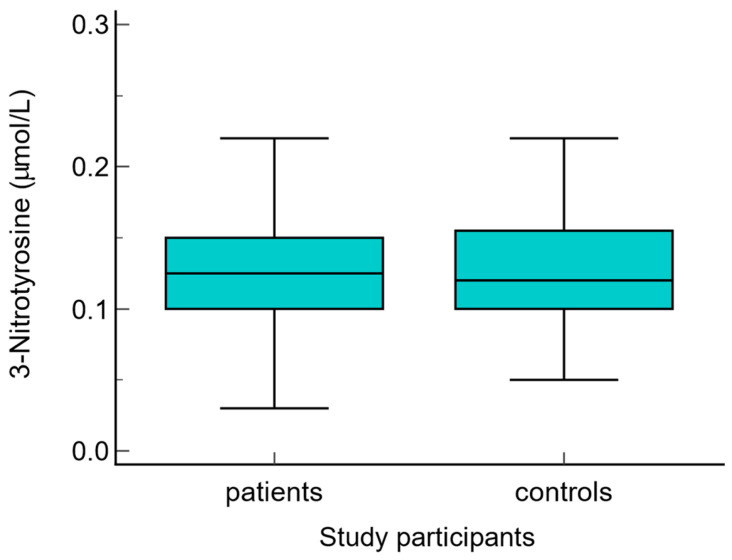
Serum levels of 3-nytrotyrosine in patients with warts and controls. The graph illustrates the distribution of the dataset, showing the median, interquartile range (middle 50% of the data), minimal, as well as maximal values, *p* > 0.05.

**Figure 8:**
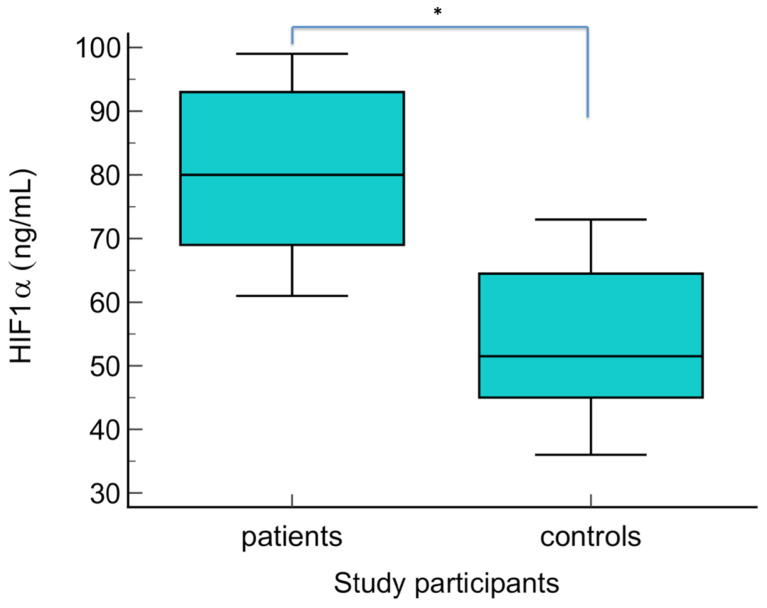
Serum levels of HIF1α in patients with warts and controls. The graph illustrates the distribution of the dataset, showing the median, interquartile range (middle 50% of the data), minimal, as well as maximal values; * indicates statistically significant difference compared to the control group, *p* < 0.01.

The serum levels of NO metabolites (direct nitrite, total nitrite, nitrate, and direct nitrite/nitrate) were statistically significantly higher in patients with warts compared to the control group (*p* < 0.01) (Figure 3, Figure 4, Figure 5 and Figure 6 Regarding the serum levels of 3-nitrotyrosine, there were no significant differences between patients with warts and controls (*p* = 0.06) (Figure 7). The serum levels of HIF1α were significantly higher in patients with warts compared to controls (p<0.01) (Figure 8).

**Figure 9:**
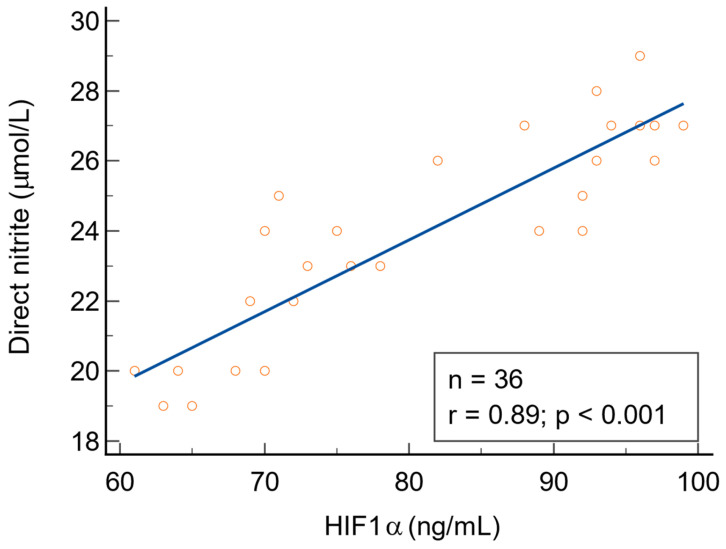
The relationship between direct nitrite and HIF1α, n = number of patients, r = 0.89 indicates a strong positive correlation, statistically significant (*p* < 0.001).

**Figure 10:**
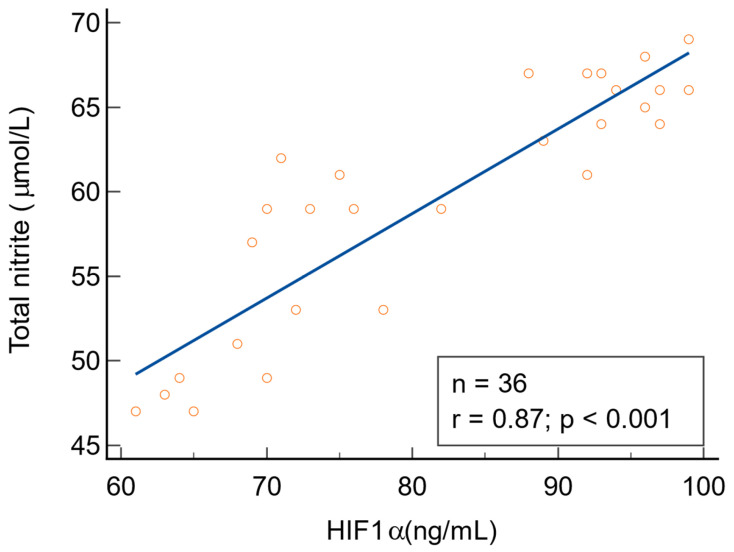
The relationship between total nitrite and HIF1α, n = number of patients, r = 0.87 indicates a strong positive correlation, statistically significant (*p* < 0.001).

**Figure 11:**
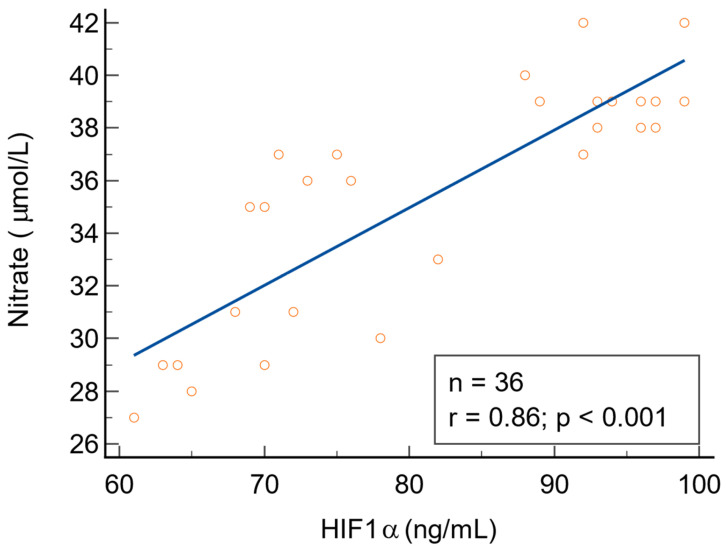
The relationship between nitrate and HIF1α, n = number of patients, r = 0.86 indicates a strong positive correlation, statistically significant (*p* < 0.001).

There were statistically significant positive correlations between NO metabolites (direct nitrite, total nitrite, and nitrate) and HIF1α in patients with warts (*p* < 0.001). There was no correlation between 3-nitrotyrosine and HIF1α (r = 0.34, *p* = 0.061). The direct relationship between NO metabolites and HIF1α is depicted in Figure 9, Figure 10 and Figure 11. We found no statistically significant correlations between NO metabolites and HIF1α in patients without warts.

There were no correlations between the serum levels of the studied parameters and the number of lesions and the duration of the disease, respectively.

## 4. Discussion

Palmoplantar warts are very common in the general population, the most affected being children, with the percentage reaching up to 33%. Subsequently, the incidence decreases with age. Given that immunity plays an important role, the percentage increases among those immunosuppressed to 45% [39]. In palmoplantar warts, HPV types 1, 2, and 4 have been identified. In the case of immunosuppressed patients, other types such as HPV 75, 76, and 77 have also been detected in the lesions. It should be noticed that many types can be detected per lesion, most commonly in the subjects with an impaired immune system [40].

The pathogenic mechanisms involved in HPV infection are incompletely elucidated. In recent decades, several studies have been published attesting to the presence of an increased level of oxidative stress in patients with warts. The studies were conducted in both tissue and serum. Arican et al. analyzed markers of oxidative stress in patients with plantar warts. They determined the levels of superoxide dismutase (SOD), catalase (CAT), and malondialdehyde (MDA) in the affected tissue compared to healthy skin samples and showed higher levels of these markers in warts [41]. These results could be explained by the tissue damage caused by HPV and the inflammatory process triggered by the presence of the virus. In line with this, Erturan et al. analyzed various oxidative stress markers in patients with genital and non-genital warts. They evaluated total antioxidant status, total oxidative status, 8-deoxy-guanosine, and thiol-disulfide homeostasis in the patient serum and identified an imbalance between oxidants and antioxidants in patients with warts compared to healthy individuals [42].

Data on the role of nitrosative stress in warts are scarce. However, there are data showing the role of NO in the case of patients with genital lesions. There are studies that support the role of NO in carcinogenesis given that high levels of NO can lead to mutations in DNA structure and alteration in p53 expression [27]. Rahkola et al. suggest that NO is an important cofactor of HPV in the occurrence of cervical cancer. Increased levels of NO in cervical fluid correlate with the persistence of hrHPV. The most important HR-HPVs are HPV 16 and HPV 18 [43]. In line with this, the study conducted by Wei et al. pointed out that NO can act as an inducer of early viral transcription These results suggest that NO is an important player in the carcinogenesis associated with HPV infection, and therapies that target this molecule could be useful in cervical cancer [20]. A recent study suggests a link between *NOS3* gene and HPV infection, but its association with cancer remains uncertain [44]. In addition, HR-HPV types have the ability to stimulate eNOS and iNOS activity in the endothelial cells and basal squamous epithelial cells of the uterine cervix [45].

One of the major features of patients with warts, proven in our study, is the increase in NO production in hypoxic conditions. This finding is supported by the following results obtained in the current paper: (1) the increase in serum levels of HIF1α, a marker of hypoxia, in patients with warts compared to controls; (2) dysregulation of NO metabolism in HPV infection through overproduction of NO and increased serum levels of nitrites, nitrates, and 3-nitrotyrosine in patients with warts versus controls; and (3) NO-HIF1α interdependence, suggested by statistically significant positive correlations between NO metabolites and HIF1α levels.

In our study, HIF1α was overregulated in patients with warts. HIF1α is a transcriptional activator that responds to oxygen levels and is involved in angiogenesis (new vessel formation). HIF1 is made up of a constantly present HIF1beta subunit and one of three possible alpha subunits (HIF1α, HIF2alpha, or HIF3alpha). HIF1α expression is modulated by several post-translational modifications such as hydroxylation, acetylation, and phosphorylation. Consequently, HIF1α activity is linked to a group of proteins such as prolyl hydroxylase domain proteins (PHD), the von Hippel–Lindau tumor suppressor protein (pVHL), ARD-1, and p300/CBP. In normal oxygen conditions, the HIF1α subunit is metabolized through the pVHL-mediated ubiquitin–proteasome pathway [46]. 

The supply of oxygen from blood vessels to the skin is limited to the dermis and subcutaneous layer, and the hypoxic microenvironment of the epidermis can lead to activation of HIF and other hypoxia pathways [47,48]. Numerous studies have reported a close bidirectional relationship between viral infections and hypoxia. The mechanisms by which HIFs modulate human viral infections include promoting transcriptional changes and regulating oxidative phosphorylation (HIF1 modulates the isoform of cytochrome c oxidase subunit 4), for more efficient use of available oxygen levels [49]. Instead, the strategies by which HPV modulates HIF1 reside in (a) the ability of HPV-16 oncoproteins to stabilize HIF1α without increasing HIF1α mRNA levels, (b) the involvement of oncoproteins E6 and E7 in promoting angiogenesis via a HIF-1α/VEGF pathway and (c) the ability of several LR-HPV and HR-HPV types to improve HIF1α expression in keratinocytes under hypoxic conditions by stabilizing HIF1α without involving PI3/mTOR, or VHL [47,49,50]. The data outlined above suggest that HIF1 modulation during HPV infection may be beneficial for disease control [50].

We found significantly higher serum levels of nitrites and nitrates in patients with warts versus controls (*p* < 0.01). Nitric oxide (NO) is a versatile molecule produced ubiquitously in mammalian cells [51,52]. Our results prove that abnormal synthesis of NO is involved in the pathogenesis of HPV infection. Our results are consistent with previous studies that have signaled dysregulation of NO metabolism in infections. Increased nitric oxide production is a universal response to hypoxic stress and plays a key role in stress signaling in microorganisms [53,54]. NO is the most important molecular cofactor generated by epithelial cells in viral infection. Under conditions of hypoxia and ischemia, endogenous NO production from L-arginine is compromised, while NOS-independent NO generation is stimulated [55]. The nitrate-nitrite-NO pathway is involved in regulating cellular metabolism, signaling, and tissue protection during hypoxia, and is designated as a hypoxia tolerance mechanism [54,56]. Increased iNOS activity and expression may represent an alternative pathophysiological mechanism to compensate for the effects of hypoxia in tissues [34]. The most important metabolites of NO are nitrites and nitrates. Thus, in order to study NO levels, it is recommended to measure the levels of these metabolites.

3-nitrotyrosine-containing proteins that result from a reaction mediated by peroxynitrite or other potential nitration agents have been identified in patients with warts. The generation of tyrosine-nitrated proteins represents a sign of the endogenous activity of peroxynitrite, and, at the same time, it should be taken into account that these compounds have been identified as playing the role of neoantigens leading to the formation of autoantibodies that can be involved in autoimmune processes [57]. In our study, 3-nitrotyrosine showed a moderate increase in patients versus controls, without statistical significance.

Altered mitochondrial respiration is one of the major events during hypoxia associated with stimulation of the nitrate-nitrite-NO axis. NO is the most important molecular cofactor secreted by HPV-infected epithelial cells [55]. The participation of NO in the development of warts can be explained by the tropism of HPV for keratinocytes and the characteristic of the skin to harbor the entire set of genes responsible for the (1) synthesis, conversion, and degradation of NO and (2) turnover and storage of NODs. Keratinocytes constitutionally express nNOS, fibroblasts and endothelial cells constantly express eNOS, and under infectious and inflammatory conditions, all skin cells and immune cells are able to express iNOS [58]. The NO production of the whole organism comes from the (1) L-arginine-NO-oxidative pathway, in which NOSs convert L-arginine to NO; (2) reductive pathway NO_3_^–^-NO_2_^–^-NO, in which NO is produced by serial reduction of nitrates (endogenous or exogenous sources) and nitrites; and (3) the conversion of NODs stored in the skin. NO catabolism involves a cycle of cytochrome c oxidase-catalyzed reactions, whereby NO is converted to nitrate and subsequently recycled or excreted in urine [6,7,59]. Under conditions of acidosis, hypoxia, and ischemia, oxidative production of endogenous NO from L-arginine is inhibited [60]. In contrast, reductive NO_3_^–^-NO_2_^–^-NO activity is increased [58].

There is also evidence that cutaneous NODs are responsible for the generation of NO, independent of NOSs, and may exert specific local and systemic effects (antimicrobial activity, suppression of acute and chronic inflammation) [8,10]. NO and NODs are highly reactive molecules that, together with other free radicals, promote the synthesis of new RNS, which through oxidation and/or nitrosylation alter the function and structure of various biomolecules [58].

In patients with warts, we found positive correlations between the serum levels of NO metabolites and HIF1α. Many NO-mediated cellular responses are known to be regulated by HIF1, and vice versa. It has been shown that NO can mediate the stabilization of HIF1α by two mechanisms, either directly by S-nitrosylation or indirectly by blocking procollagen-proline dioxygenase activity. That demonstrates the existence of an interdependence between NO and HIF1α. Increased levels of NO induce the expression of HIF1α and its accumulation, while when the level of NO is low, the level of HIF1α also decreases [47,49,61,62]. In turn, HIF1 increases NO production through overexpression of iNOS and activation of cytochrome c oxidase subunit4 isoform2 (COX4-2) [47,63]. Increased amounts of NO produced by iNOS mitigate the stabilization of HIF1alpha under hypoxic conditions by competing with O_2_. During hypoxia, NO inhibits the accumulation of HIF1α. In contrast, under normoxic conditions, NO has been shown to induce increased levels of HIF1α and its activity. The property of NO to mimic hypoxic signaling suggests that NO and hypoxia might utilize similar pathways to stabilize HIF1α. It has been shown that the induction of HIF1α protein by NO takes place independently of the traditional NO signaling pathway, through soluble guanylyl cyclase (sGC) and cyclic GMP (cGMP) [61,64].

These observations explain the positive correlations we obtained between serum levels of NO metabolites and serum HIF1α levels. Consequently, NO-HIF mutual signaling could influence processes such as angiogenesis, apoptosis, and senescence [47,49,61,63]. We found no statistically significant correlations between NO metabolites and HIF1α in patients without warts. This may be explained by the fact that the positive correlations between NO metabolites and HIF1α in patients with warts reflect the pathogenic mechanisms involved in HPV infection such as chronic inflammation and hypoxia, that are absent in subjects without warts. In patients with warts, in the context of the inflammation and hypoxia induced by HPV, the high levels of NO facilitate HIF1α production, and this is why there are correlations between NO and HIF1α as we discussed above.

However, there was no correlation between the serum levels of HIF1α and 3-nitrotyrosine. The lack of correlation between serum levels of HIF1α and 3-nitrotyrosine in patients with HPV infection could be explained by the following factors: (1) hypoxia mediates nitrosative stress; (2) although 3-nitrotyrosine has been revealed as a relevant biomarker of NO-dependent oxidative stress, tyrosine nitration is a low-efficiency process in vivo; (3) several nitration pathways may contribute to tyrosine nitration in vivo; (4) most of the nitration pathways involve the presence of free radicals (peroxynitrite anion, nitrogen dioxide, superoxide radicals, hydrogen peroxide), transitional metals, or hemepoxidase [65,66].

In this study, we also analyzed the relationship between disease duration and the number of warts on one hand and the markers of NO metabolism and hypoxia on the other. There were no correlations between serum levels of the parameters studied and the number of lesions and the duration of the disease, respectively.

In this work, dysregulation of NO metabolism has been reported in patients with warts, but it is not known whether patients recovered from the disease have restored NO. In further studies, we will assess the NO status after HPV infection. Low NO_2_^−^/NO_3_^−^ and high NO_3_^−^ can be potential biomarkers of poor or irreversible long-term outcomes after SARS-CoV-2 infection. It suggests that NO metabolites could serve as a predictor of the health status of recovered COVID-19 patients, highlighting the need to elucidate the role of NO after viral infections [67].

There is a requirement for additional research to enhance our comprehension of the origins and targets of RNS, their potential functions, the precise molecular pathways involved, their interactions, and to pinpoint the optimal patient population that may respond favorably to antioxidant treatments. Understanding the role of nitrosative/oxitive stress in warts can serve as the foundation for the development of new therapies, namely antioxidant treatments. Antioxidant therapy has gained significant attention. Antioxidant systems are involved in repairing alterations caused by the action of RNS/ROS on various cellular components [68]. The research on targeted antioxidant therapy could lead to a significant advancement in the treatment of dermatological diseases, but additional studies are needed to improve our understanding of how oral or topical antioxidant agents may improve the course of some dermatological afflictions [69].

Our research could provide new insights for a comprehensive understanding of the pathogenesis of HPV infection. Our findings could also be useful for initiating further research into the pathogenesis of HPV infection. Also, the results could be exploited to design therapeutic strategies. This is the first study to report the interaction between hypoxia and NO in patients with warts. The limitations of the research were the small number of cases and that the study was conducted in a single center. Another limitation of the study is that we did not identify the HPV types involved in the appearance of warts. Our results need to be confirmed in larger studies using cell lines to better understand the pathogenic mechanisms involved in the dysregulation of NO homeostasis in patients with warts.

## 5. Conclusions

In patients with warts, we found serum variations in NO metabolites depending on the degree of hypoxia. Overproduction of NO is a pathological event during HPV infections. Increased NO synthesis could be due to overexpression of iNOS under hypoxic conditions and/or stimulation of nitrate-nitrite-NO molecular pathway. In HPV infection there is a mutual modulation of NO-hypoxia responses which suggests the close link between NO metabolism and hypoxia.

## Data Availability

All data are contained within the article.

## Abbreviations

CVB	coxsackievirus
HCV	hepatitis C virus
HBV	hepatitis B virus
FAD	flavin adenine dinucleotides
FMN	flavin mononucleotides
BH4	tetrahydrobiopterin
NADP+	nicotinamide adenine dinucleotide phosphate
NADPH	reduced nicotinamide adenine dinucleotide phosphate
S-GSNO	S-nitrosoglutathione
HIV	human immunodeficiency virus
HPV	human papillomavirus
LR-HPVs	low-risk human papillomaviruses
HR-HPVs	high-risk human papillomaviruses
HSV	herpes simplex virus
PCV2	porcine circovirus type 2
RSV	respiratory syncytial virus
SARS-CoV-2	severe acute respiratory syndrome coronavirus 2
NODs	nitric oxide derivates
RSNO	S-nitroso compounds
RNNO	N-nitroso compounds
ONOO	peroxynitrite
NO	nitric oxide
NOS	nitric oxide synthase
eNOS	endothelial nitric oxide synthase
nNOS	neuronal nitric oxide synthase
iNOS	inducible nitric oxide synthase
NO_2_^−^	nitrites
NO_3_^−^	nitrates
COX4-2	cytochrome c oxidized subunit4 isoform2
PHDs	prolyl-hydroxylases
HIF1/2	hypoxia-inducible factor 1/2
OS	oxidative stress
ROS	reactive oxygen species
RNS	reactive nitrogen species
